# Case of Fungus from the Synovial Capsule of the Knee, under the Care of M. Gerdy, of the Hôpital De La Charite, Treated by Compression

**Published:** 1845-01

**Authors:** 


*Case of Fungus from the Synovial Capsule of the Knee, under the care of* M. Gerdy, *of the Hôpital de la Charite, treated by Compression.*—The subject of this case is a young man, aged 30, of a lymphatic and deteriorated constitution. On the internal surface of the knee there is an ulcer of the size of the palm of the hand, from whose base has sprung an ill-conditioned fungus of the size of half an orange, and which, according to M. Gerdy, originates from the external surface of the synovial capsule, and has no communication with the joint. The articular movements are quite free, and cause no discharge of pus, as would be the case if the disease communicated with the synovial cavity. In M. Gerdy's opinion, this is the best test that can be employed for the purpose of ascertaining if a tumour containing fluid, and which has been opened, communicates with the neighboring articulation or not. The patient has an affection of the same nature on the dorsal surface of the wrist. The fungus neither appears cancerous, nor has any tendency to bleed: at the knee, as well as at the wrist, it is composed of an enormous number of granulations of the size of a nut, having a greyish colour, and of a soft, dirty aspect, and yielding a large quantity of fetid pus. The means employed by M. Gerdy for the treatment of this affection are, compression by means of strips of adhesive plaster, and under the influence of these, and rest, the disease is improving. M. Gerdy has met with one other case of this rare disease; it was situate at the elbow-joint, and occurred in an individual in whom he had amputated the leg, but who refused to submit to the loss of his arm; it was treated by compression, and a cure followed, but there was anchylosis of the joint.

In a pathological point of view, this is a rare affection; indeed, it is the first time we have met with it. An attentive examination of the joint leads to the idea that there is hypertrophy of the whole synovial capsule, with tendency to fungus from its external surface. It appears to us that the affection may be compared to certain non-cancerous fungi which occur on the mucous membrane of the alveolar processes, and which have been denominated epuli. Its co-existence at the knee and wrist at one and the same time, and the deteriorated state of the constitution, would lead us to suspect that it originates from some peculiar diathesis. There are, however, no distinct signs of scrofula, although the patient has a pale cachectic look; but this appearance is, perhaps, an effect of the local disease itself. However this may be, M. Gerdy has commenced the treatment by compression, to which he proposes to add the internal use of the iodide of potassium. A marked diminution in the size of the vegetations has already taken place, under the use of the compression alone.—*Ibid, from Annales de Therapeutique.*

